# Interleukin‐38 suppresses abdominal aortic aneurysm formation in mice by regulating macrophages in an IL1RL2‐p38 pathway‐dependent manner

**DOI:** 10.14814/phy2.15581

**Published:** 2023-01-28

**Authors:** Shun Kurose, Yutaka Matsubara, Shinichiro Yoshino, Keiji Yoshiya, Koichi Morisaki, Tadashi Furuyama, Tomoaki Hoshino, Tomoharu Yoshizumi

**Affiliations:** ^1^ Department of Surgery and Science, Graduate School of Medical Sciences Kyushu University Fukuoka Japan; ^2^ Department of Kidney Center Saiseikai Yahata General Hospital Fukuoka Japan; ^3^ Division of Respirology, Neurology and Rheumatology, Department of Medicine Kurume University School of Medicine Fukuoka Japan

**Keywords:** abdominal aortic aneurysm, inflammation, Interleukin‐38, macrophage

## Abstract

Macrophages play crucial roles in abdominal aortic aneurysm (AAA) formation through the inflammatory response and extracellular matrix degradation; therefore, regulating macrophages may suppress AAA formation. Interleukin‐38 (IL‐38) is a member of the IL‐1 family, which binds to IL‐36 receptor (IL1RL2) and has an anti‐inflammation effect. Because macrophages express IL1RL2, we hypothesized that IL‐38 suppresses AAA formation by controlling macrophages. We assessed a C57BL6/J mouse angiotensin II‐induced AAA model with or without IL‐38 treatment. RAW 264.7 cells were cultured with tumor necrosis factor‐α and treated with or without IL‐38. Because p38 has important roles in inflammation, we assessed p38 phosphorylation in vitro and in vivo. To clarify whether the IL‐38 effect depends on the p38 pathway, we used SB203580 to inhibit p38 phosphorylation. IL1RL2^+^ macrophage accumulation along with matrix metalloproteinase (MMP)‐2 and ‐9 expression was observed in mouse AAA. IL‐38 reduced the incidence of AAA formation along with reduced M1 macrophage accumulation and MMP‐2 and ‐9 expression in the AAA wall. Macrophage activities including inducible nitric oxide, MMP‐2, and MMP‐9 production and spindle‐shaped changes were significantly suppressed by IL‐38. Furthermore, we revealed that inhibition of p38 phosphorylation diminished the effects of IL‐38 on regulating macrophages to reduce AAA incidence, indicating the protective effects of IL‐38 depend on the p38 pathway. IL‐38 plays protective roles against AAA formation through regulation of macrophage accumulation in the aortic wall and modulating the inflammatory phenotype. Using IL‐38 may be a novel therapy for AAA patients.

## Graphical abstract

Interleukin‐38 (IL‐38) reduced the incidence of abdominal aortic aneurysm (AAA) formation in angiotensin II‐induced C57BL6/J mice. IL‐38 suppressed macrophage accumulation and MMP‐2 and ‐9 expression in the aortic wall during AAA formation. The protective effects of IL‐38 against AAA formation depend on the p38 pathway.

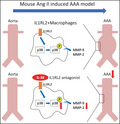

## 1 INTRODUCTION

Abdominal aortic aneurysm (AAA) is a life‐threatening disease associated with a high mortality rate after rupturing; therefore, AAA should be treated before rupture (Hoornweg et al., 2008). Currently, only surgical interventions can prevent AAA rupture. Non‐surgical medical treatments, such as an angiotensin‐converting enzyme inhibitor and an angiotensin receptor blocker, have been proposed; however, these treatments did not show significant evidence of preventing AAA progression or rupture (Bicknell et al., 2016; Thompson et al., 2010). Because surgical interventions are sometimes too invasive for patients with severe comorbidities or advanced age, less invasive treatments are required. To develop such alternative treatments, there is an urgent need to understand potential therapeutic targets.

The key mechanisms of AAA formation are inflammation and extracellular matrix (ECM) degradation of the aortic wall (Ailawadi et al., 2003). Macrophages play crucial roles in the production of inducible nitric oxide (iNOS) and matrix metalloproteinases (MMPs), which cause inflammation and ECM degradation, resulting in the formation and progression of AAA (Dale et al., 2015; Raffort et al., 2017).

Interleukin‐38 (IL‐38) is a member of the IL‐1 family, which binds to IL‐36 receptor (IL1RL2) as an antagonist to exert an anti‐inflammation effect (Esmaeilzadeh et al., 2021; Lai et al., 2022; van de Veerdonk et al., 2012). Also, IL1RL2 was reported as a candidate gene‐related soluble ST2 those elevated levels reflected the degree of vascular injury (Jiang et al., 2021). Because IL1RL2 controls the inflammatory response (Buhl & Wenzel, 2019; Mahil et al., 2017), IL‐38 can suppress inflammatory cells. Previous reports have shown that IL‐38 exerts protective functions against various inflammatory diseases such as inflammatory bowel disease (Ohno et al., 2022), psoriasis (Han et al., 2019), encephalomyelitis (Huard et al., 2021), asthma (Matsuoka et al., 2019; Sun et al., 2020), cardiac remodeling (Wei et al., 2020), and sepsis (Ge et al., 2020; Ge et al., 2021). IL‐38 restrains the inflammatory response by regulating macrophages (Ge et al., 2021; Mora et al., 2016; Xie et al., 2020). Although the association between IL‐38 and AAA has not been clarified, IL‐38 may have important roles in regulating inflammatory macrophages. We, therefore, hypothesized that IL‐38 regulates macrophages to control inflammation and ECM degradation of the aortic wall in AAA formation.

## 2 MATERIALS AND METHODS

### 2.1 Experimental approval

All animal experiments were approved by the Animal Care and Use Committee of Kyushu University (Approval No. A21‐347‐2) and all animals were handled in accordance with nationally prescribed guidelines and Animal Research Reporting of In Vivo Experiments (ARRIVE) guidelines.

### 2.2 Cell culture

RAW 264.7 cells, a mouse macrophage cell line, were purchased from American Type Culture Collection (ATCC® #TIB‐71™). Cells were cultured in a CO_2_ incubator at 37°C and 5% CO_2_ in Dulbecco's modified Eagle medium (DMEM; Gibco; Thermo Fisher Scientific) supplemented with 10% fetal bovine serum (FBS), penicillin (100 U/ml), and streptomycin (100 mg/ml). Cells were passaged upon reaching 80%–90% confluence and seeded into a 6‐well plate at a density of 5 × 10^5^/ml for further experiments. Cells between 3 and 15 passages were used in the experiments. In vitro, RAW 264.7 cells were incubated with tumor necrosis tumor necrosis factor‐α (TNF‐α; #410‐MT; R&D systems) for 24 h with 0, 1, 5, 10, 20, and 50 ng/ml to determine the optimal concentration for macrophage activation.

### 2.3 Morphological observation of macrophages

The morphology of macrophages was observed using an Olympus BX51 microscope (Olympus) and the percentage of spindle‐shaped macrophages out of the total was examined (Soliman et al., 2022). Briefly, we counted macrophages and calculated the percentage of the spindle‐shaped macrophages per high‐power field (HPF), then the mean percentage in each well was determined by averaging the values of five HPFs.

### 2.4 Mouse abdominal aortic aneurysm model

Wild‐type male C57BL6/J mice were purchased from The Jackson Laboratory. They were kept on a 12/12 h light/dark cycle and fed standard chow (CLEA) and water ad libitum. At 10–12 weeks of age the mice were used for animal experiments. Surgical procedures were performed under general anesthesia. Briefly, mice were anesthetized in a closed chamber with 1.5% isoflurane in oxygen for several minutes until immobile. Each mouse was fixed by tape on a heated (37°C) procedure board with 1.5% isoflurane administered via a nosecone during the following surgery. Mini osmotic pumps (Model 2004; Alzet) containing angiotensin II (Ang II; #A9525; Sigma–Aldrich) with an infusion rate of 1000 ng/kg/min were implanted subcutaneously in the neck region of anesthetized mice. The mice were observed daily after surgical procedures. The tissues were harvested after euthanasia on days 0, 3, 7, and 14 after pump implantation. Necropsy was performed to confirm the cause of death as soon as an animal expired before harvesting. Considering tissue degradation, these animals were excluded from the histological analysis but used for AAA incidence and mortality data.

### 2.5 IL‐38 and SB203580 treatments

Mouse recombinant IL‐38 protein was provided by Kurume University (Matsuoka et al., 2019). The dosage and administration of IL‐38 were according to previous reports (Ge et al., 2021; Wei et al., 2020).

RAW 264.7 cells were treated with IL‐38 (100 ng/ml) or the same amount of phosphate‐buffered saline (PBS) 30 min before TNF‐α (#410‐MT; R&D systems) stimulation in the in vitro experiments.

In vivo, 1.0 μg of IL‐38 was intraperitoneally administrated just before and twice a week after pump implantation until tissue harvest. The same amount of PBS (200 μl) was used as a control.

SB203580 (#S1076; Selleck) was dissolved in 1% dimethyl sulfoxide (DMSO) at 1 mg/ml. RAW 264.7 cells were treated with SB203580 (10 μM) 60 min before TNF‐α stimulation. Mice were injected intraperitoneally with SB203580 (10 mg/kg) or 1% DMSO daily for 14 days, starting 30 min before the pump implantation.

### 2.6 Histological analysis

The aortas were fixed with 10% formalin for 24 h, embedded in paraffin, and cut into 4‐μm cross sections. Hematoxylin & eosin (H&E) staining and Elastica Van Gieson staining were performed for histological assessments. Stained slides were scanned and analyzed using NanoZoomer (Hamamatsu Photonics KK). The abdominal aortic diameter was calculated as external circumferential length/pi. AAA formation was defined by the expansion of the abdominal aorta by 150% of the aortic diameter compared with day 0. The AAA incidence rate included AAA formation and death of rupture in mice. The areas surrounded by the luminal surface, external elastic lamina, and external circumference were measured. The elastin degradation score was graded as previously described: grade 1—no degradation and a well‐organized elastin lamina; grade 2—mild degradation; grade 3—severe degradation; and grade 4—presence of aortic rupture (Song et al., 2022).

### 2.7 Immunohistochemistry

Immunohistochemistry was performed on 4‐μm formalin‐fixed and paraffin‐embedded sections. Tissue sections were de‐paraffined using xylene and a graded ethanol series. For antigen retrieval, sections were heated in citric acid buffer (pH 6.0 or 9.0) at 121°C for 15 min according to the manufacturer's protocol. Nonspecific background endogenous peroxidase activity was blocked by pre‐treatment with 3% hydrogen peroxide in methanol for 30 min. Sections were blocked with 10% bovine serum for 1 h at room temperature, and then incubated at 4°C with the primary antibody overnight: anti‐ TNF‐α (rabbit polyclonal, dilution 1:100, #ab6671; abcam), anti‐MMP‐9 (rabbit polyclonal, dilution 1:1000, #ab38898; abcam), anti‐ MMP‐2 (rabbit polyclonal, dilution 1:1000, #ab97779; abcam), anti‐IL1RL2 (rabbit polyclonal, dilution 1:200, # PA5‐38013; Invitrogen) and anti‐CD68 (rat monoclonal, dilution 1/100, MCA1957; Bio‐Rad). After incubation, the sections were incubated with Dako EnVision® + Systems‐HRP (K4003; Dako [Agilent Technologies, Inc.], Santa Clara, CA, USA) for 1 h at room temperature and treated with 3,3′‐diaminobenzidine to detect the reaction products. Finally, the sections were counterstained with hematoxylin and mounted on glass slides. We counted the total number of positive cells in the abdominal aortic wall cross section and defined the value divided by the aortic wall area (mm^2^) as the number of positive cells (/mm^2^).

### 2.8 Immunofluorescence

Tissue sections were de‐paraffined, and then heated in citric acid buffer (pH 6.0) at 121°C for 15 min for antigen retrieval. The sections were blocked with 10% bovine serum before incubation with primary antibodies overnight at 4°C: anti‐CD68 (rat monoclonal, dilution 1/100, MCA1957; Bio‐Rad), anti‐IL1RL2 (rabbit polyclonal, dilution 1:200, # PA5‐38013; Invitrogen), anti‐TNF‐α (rabbit monoclonal, dilution 1:400, #11948; Cell Signaling Technology), anti‐iNOS (rabbit monoclonal, dilution 1:400, #13120; Cell Signaling Technology), anti‐Arginase‐1 (rabbit monoclonal, dilution 1:400, #93668; Cell Signaling Technology), and anti‐CD206 (rabbit polyclonal, dilution 0.1 μg/ml, ab64693; Abcam). After incubation, the sections were incubated with Alexa Fluor 488‐conjugated (A11006, 5 μg/ml; Invitrogen) or Alexa Fluor 594‐conjugated (A11012, 5 μg/ml, Invitrogen) secondary antibodies for 1 h, and then mounted with 4′,6‐diamidino‐2‐phenylindole (DAPI) for 10 min at room temperature. Cells were observed using a fluorescence microscope (Biorevo BZ‐90000; Keyence).

### 2.9 Western blotting

Samples were lysed in lysis buffer containing 50 mmol/L Tris–HCl (pH 6.8) and 10% sodium dodecyl sulfate, and the protein concentration of each sample was determined using a Bio‐Rad Protein Assay kit (Bio‐Rad Laboratories). Samples were heated at 95°C for 5 min and subjected to electrophoresis using SuperSep Ace 10%–20% gels (Fujifilm) at 20 mA for 80 min. The Trans‐Blot Turbo Transfer System (Bio‐Rad) was used to transfer proteins onto a polyvinylidene fluoride membrane (Bio‐Rad). The membrane was incubated consecutively in iBind solution (Invitrogen) containing primary and secondary antibodies. Primary antibodies included anti‐TNF‐α (rabbit monoclonal, dilution 1:400, #11948; Cell Signaling Technology), anti‐iNOS (rabbit monoclonal, dilution 1:400, #13120; Cell Signaling Technology), anti‐ MMP‐9 (rabbit polyclonal, dilution 1:1000, #ab38898; abcam), anti‐ MMP‐2 (rabbit polyclonal, dilution 1:1000, #ab97779; abcam), anti‐IL1RL2 (rabbit polyclonal, dilution 1:1000, # PA5‐38013; Invitrogen), anti‐p38 (rabbit polyclonal, dilution 1:5000, #9212; Cell signaling technology), anti‐phospho‐p38 (p‐p38) (rabbit polyclonal, dilution 1:1000, #9211; Cell signaling technology), anti‐JNK (rabbit polyclonal, dilution 1:5000, #9252; Cell signaling technology) and anti‐phospho‐JNK (p‐JNK) (rabbit polyclonal, dilution 1:1000, #9251; Cell signaling technology).

### 2.10 Statistical analysis

Data are represented as mean ± SEM. All data were analyzed using Prism 9 software (GraphPad Software, Inc.). Normality was confirmed using the Shapiro–Wilk test. Statistical significance was determined using Student's *t* test or ANOVA with Tukey's post hoc correction. We used the Mann–Whitney U test or the Kruskal–Wallis test with Dunn's post hoc correction if the sample size was smaller than 6. *p* < 0.05 was considered statistically significant.

## 3 RESULTS

### 3.1 IL1RL2^+^ macrophages accumulate in the aortic wall of C57BL6/J mice along with TNF‐α, MMP‐9, and MMP‐2 upon angiotensin II infusion

**FIGURE 1:**
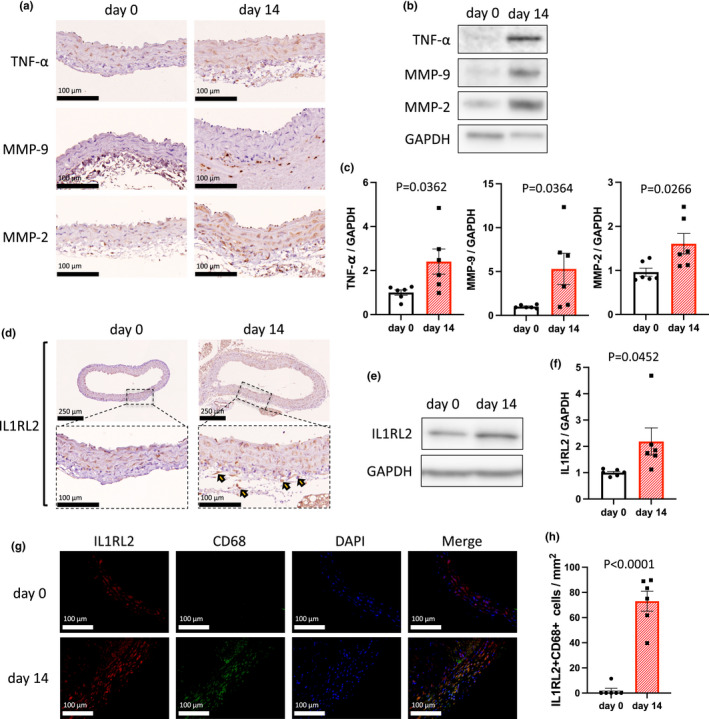
IL1RL2^+^ macrophages accumulated in the mouse abdominal aortic wall after angiotensin II infusion. (a) Representative photomicrographs showing the abdominal aortic wall on days 0 and 14 stained with anti‐TNF‐α, anti‐MMP‐9, or anti‐MMP‐2 antibody. Scale bar, 100 μm. (b) Representative western blot analysis of TNF‐α, MMP‐9, MMP‐2, and GAPDH immunoreactivity in the abdominal aortic wall on days 0 and 14. (c) Bar graphs showing the relative densitometry of TNF‐α (*n* = 6, *p* = 0.0362 [*t* test], *p* = 0.0260 [Mann–Whitney U test]), MMP‐9 (*n* = 6, *p* = 0.0364 [*t* test], *p* = 0.0152 [Mann–Whitney U test]), and MMP‐2 (*n* = 6, *p* = 0.0266 [*t* test], *p* = 0.0260 [Mann–Whitney U test]). (d) Representative photomicrographs showing the abdominal aortic wall stained with anti‐IL1RL2 antibody on days 0 and 14. Arrowheads show representative IL1RL2^+^ cells. Scale bar, 100 μm. (e) Representative western blot analysis of IL1RL2 and GAPDH immunoactivity in the abdominal aortic wall on days 0 and 14. (f) Bar graph showing the relative densitometry of IL1RL2. *n* = 6, *p* = 0.0452 (*t* test), *p* = 0.0022 (Mann–Whitney U test). (g) Representative photomicrographs showing IL1RL2 (red) and CD68 (green) in aortic tissue on days 0 and 14. Scale bar, 100 μm. (h) Bar graph showing the number of IL1RL2^+^CD68^+^ cells in aortic tissue on days 0 and 14. *n* = 6, *p* < 0.0001 (*t* test), *p* = 0.0022 (Mann–Whitney U test).

Because TNF‐α has a key role for AAA formation (Puchenkova et al., 2022) and stimulates macrophages to produce MMP‐9 and MMP‐2 during AAA development (Xiong et al., 2009), we examined the expression of TNF‐α, MMP‐9, and MMP‐2 in the abdominal aortic wall after angiotensin II infusion for 14 days. Immunohistochemistry demonstrated the presence of TNF‐α, MMP‐9, and MMP‐2 in the abdominal aortic wall on day 14 (Figure 1a). Additionally, western blotting showed a significant increase in TNF‐α, MMP‐9, and MMP‐2 protein expression in the abdominal aortic wall on day 14 (Figure 1b,c). These findings indicated that increased TNF‐α, MMP‐9, and MMP‐2 expression were associated with AAA development in C57BL6/J mice after angiotensin II infusion.

We next focused on IL1RL2, which can regulate macrophage function (Ge et al., 2021). We performed immunohistochemistry for IL1RL2 in the mouse aorta after angiotensin II infusion. Figure 1d shows the accumulation of IL1RL2^+^ cells in the abdominal aortic wall on day 14 after angiotensin II infusion. Western blotting also showed that IL1RL2 immunoreactivity was significantly increased in the abdominal aorta on day 14 after angiotensin II infusion, compared with day 0 (Figure 1e,f). Furthermore, immunofluorescence showed that IL1RL2 was localized in CD68^+^ cells (Figure 1g,h), indicating that IL1RL2^+^ macrophages accumulate in the aortic wall of C57BL6/J mice after angiotensin II infusion. These results indicated that IL1RL2^+^ macrophages may have important roles in regulating MMP‐9 and MMP‐2 expression in AAA development.

### 3.2 IL‐38 reduces MMP‐9 and MMP‐2 production in macrophages in the aortic wall

**FIGURE 2:**
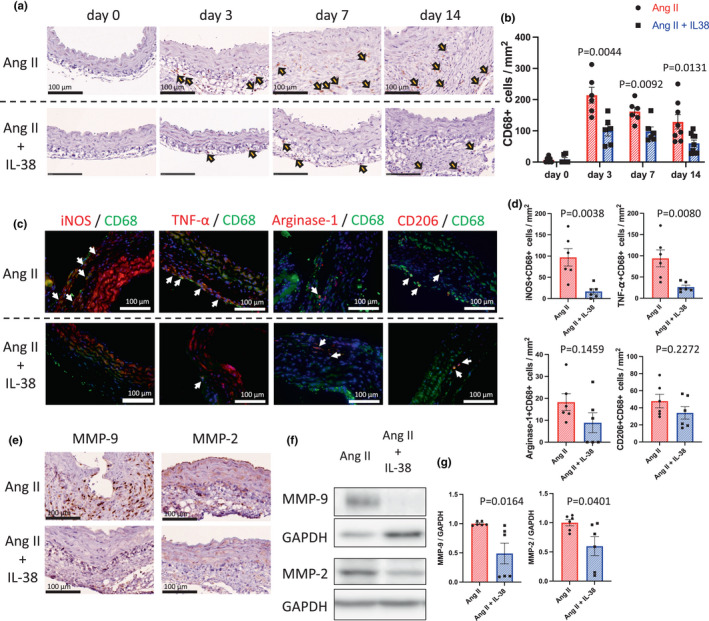
IL‐38 suppressed the accumulation and activation of macrophages in the C57BL6/J mouse aortic wall after angiotensin II infusion. (a) Representative photomicrographs of the aortic wall in mice on days 0, 3, 7, and 14, stained with anti‐CD68 antibody; upper, Ang II group; lower, Ang II + IL‐38 group. Scale bar, 100 μm. Arrowheads show representative CD68^+^ cells. (b) Bar graph showing the number of CD68^+^ cells in the aortic wall on day 0 (*n* = 6, *p* = 0.8602 [*t* test], *p* > 0.9999 [Mann–Whitney U test]), day 3 (*n* = 6, *p* = 0.0044 [*t* test], *p* = 0.0043 [Mann–Whitney U test]), day 7 (*n* = 6, *p* = 0.0092 [*t* test], *p* = 0.0152 [Mann–Whitney U test]), day 14 (*n* = 8–10, *p* = 0.0131 [*t* test], *p* = 0.0152 [Mann–Whitney U test]). (c) Representative photomicrographs showing CD68 (green) and iNOS (red), CD68 (green) and TNF‐α (red), CD68 (green) and Argnase‐1 (red), and CD68 (green) and CD206 (red) in the aortic wall of mice (day 14); upper, Ang II group; lower, Ang II + IL‐38 group. (d) Bar graphs showing the number of iNOS^+^CD68^+^ cells (*n* = 6, *p* = 0.0038 [*t* test], *p* = 0.0043 [Mann–Whitney U test]), TNF‐α^+^CD68^+^ cells (*n* = 6, *p* = 0.0080 [*t* test], *p* = 0.0087 [Mann–Whitney U test]), Arginase‐1^+^CD68^+^ cells (*n* = 6, *p* = 0.1459 [*t* test], *p* = 0.1775 [Mann–Whitney U test]), and CD206^+^CD68^+^ cells (*n* = 6, *p* = 0.2272 [*t* test], *p* = 0.3095 [Mann–Whitney U test]). (e) Representative photomicrographs showing the aortic wall of mice (day 14) stained with anti‐MMP‐9 or MMP‐2 antibody; upper, Ang II group; lower, Ang II + IL‐38 group. Scale bar, 100 μm. (f) Representative western blot analysis of MMP‐9, MMP‐2, and GAPDH immunoreactivity in the abdominal aortic wall of mice (day 14, Ang II group or Ang II + IL‐38 group). g, Bar graphs showing relative densitometry of MMP‐9 (*n* = 6, *p* = 0.0164 [*t* test], *p* = 0.0043 [Mann–Whitney U test]) and MMP‐2 (*n* = 6, *p* = 0.0401 [*t* test], *p* = 0.0260 [Mann–Whitney U test]).

To determine whether IL‐38 treatment reduces MMP‐9 and MMP‐2 production in macrophages in the aortic wall, we assessed the mouse aortic wall after angiotensin II infusion. Macrophage accumulation was observed during angiotensin II infusion with or without IL‐38 treatment by immunohistochemistry of the abdominal aortic wall for CD68 staining on day 0, 3, 7, and 14 (Figure 2a). IL‐38 treatment significantly reduced the accumulation of CD68^+^ cells on days 3, 7, and 14 (Figure 2b). Furthermore, we examined whether inflammatory M1 or anti‐inflammatory M2 macrophage accumulation was reduced by IL‐38 treatment after angiotensin II infusion. We performed immunofluorescence of the abdominal aortic wall after angiotensin II infusion to assess the presence of iNOS^+^CD68^+^ (M1), TNF‐α^+^CD68^+^ (M1), Arginase‐1^+^CD68^+^ (M2), and CD206^+^CD68^+^ (M2) cells on day 14 (Figure 2c). IL‐38 treatment significantly reduced the accumulation of iNOS^+^CD68^+^ cells and TNF‐α^+^CD68^+^ cells (M1 macrophages); however, it did not reduce the accumulation of Arginase‐1^+^CD68^+^ cells and CD206^+^CD68^+^ cells (M2 macrophages; Figure 2d). These findings indicated that IL‐38 treatment reduced inflammatory M1 macrophage accumulation in the aortic wall during AAA development while maintaining anti‐inflammatory M2 macrophages.

We also assessed the MMP‐9 and MMP‐2 expression in the aortic wall after angiotensin infusion. IL‐38 significantly reduced MMP‐9 and MMP‐2 expression in the mouse abdominal aortic wall after angiotensin infusion (Figure 2e–g). These results suggest that IL‐38 suppressed the accumulation of M1 macrophages and reduced MMP‐9 and MMP‐2 expression in the abdominal aortic wall during AAA development, indicating that IL‐38 treatment may be able to reduce AAA development.

### 3.3 IL‐38 suppresses aortic enlargement, AAA incidence, and elastin degradation in mouse angiotensin II infusion AAA model

**FIGURE 3:**
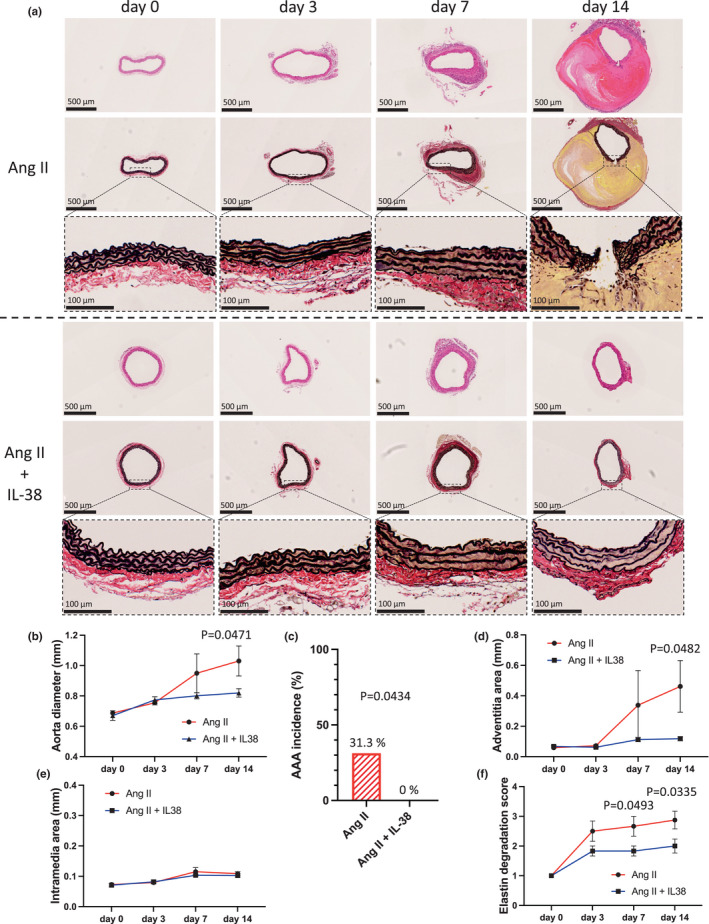
Histological changes in the abdominal aorta in C57BL6/J mice after angiotensin II infusion with or without IL‐38 treatment. (a) Representative photomicrographs of the aortic wall stained with H&E (upper) or EVG (middle and lower). (b) Line graph showing the aortic diameter on day 0 (*n* = 6, *p* = 0.6406 [*t* test], *p* > 0.9999 [Mann–Whitney U test]), day 3 (*n* = 6, *p* = 0.5051 [*t* test], *p* = 0.6688 [Mann–Whitney U test]), day 7 (*n* = 6, *p* = 0.2771 [*t* test], *p* = 0.6991 [Mann–Whitney U test]), and day 14 (*n* = 8–10, *p* = 0.0471 [*t* test], *p* = 0.0273 [Mann–Whitney U test]). (c) Bar graph showing the AAA incidence and death of rupture on days 7–14 (*p* = 0.0434 [χ^2^ test]). (d) Line graph showing the adventitia area on day 0 (*n* = 6, *p* = 0.4195 [*t* test], *p* = 0.3939 [Mann–Whitney U test]), day 3 (*n* = 6, *p* = 0.1969 [*t* test], *p* = 0.1797 [Mann–Whitney U test]), day 7 (*n* = 6, *p* = 0.3429 [*t* test], *p* = 0.6212 [Mann–Whitney U test]), and day 14 (*n* = 8–10, *p* = 0.0482 [*t* test], *p* < 0.0001 [Mann–Whitney U test]). (e) Line graph showing the intramedia area on day 0 (*n* = 6, *p* = 0.7292 [*t* test], *p* = 0.9047 [Mann–Whitney U test]), day 3 (*n* = 6, *p* = 0.7610 [*t* test], *p* = 0.7338 [Mann–Whitney U test]), day 7 (*n* = 6, *p* = 0.4324 [*t* test], *p* = 0.4567 [Mann–Whitney U test]), and day 14 (*n* = 6, *p* = 0.4689 [*t* test], *p* = 0.5246 [Mann–Whitney U test]). (f) Line graph showing the elastin degradation score on day 0 (*n* = 6, *p* > 0.9999 [*t* test], *p* > 0.9999 [Mann–Whitney U test]), day 3 (*n* = 6, *p* = 0.1099 [*t* test], *p* = 0.2727 [Mann–Whitney U test]), day 7 (*n* = 6, *p* = 0.0493 [*t* test], *p* = 0.1212 [Mann–Whitney U test]), day 14 (*n* = 6, *p* = 0.0335 [*t* test], *p* = 0.05277 [Mann–Whitney U test]).

Because IL‐38 suppressed M1 macrophage accumulation and reduced MMP‐9 and MMP‐2 expression in the aortic wall (Figure 2), we examined the effects of IL‐38 treatment on the structures of the abdominal aortic wall in mouse angiotensin II infusion AAA model. H&E staining or Elastica van Gieson staining show the histological changes of the aortic wall after angiotensin II infusion over time (Figure 3a). The aortic diameter gradually increased after angiotensin II infusion; however, IL‐38 significantly suppressed this increase (Figure 3b). Moreover, no AAA formation was observed in the IL‐38‐treated mice, while the incidence of AAA formation in the non‐treated mice after angiotensin II infusion on day 14 was 31.3% (Figure 3c). Ang II infusion initially stimulates adventitial fibroblasts to recruit and activate monocytes followed by adventitia thickening (Ijaz et al., 2017; Tieu et al., 2011), hence we assessed the adventitia and intramedia separately. The adventitia area was significantly reduced in IL‐38‐treated mice after angiotensin II infusion on day 14 (Figure 3d), although the intramedia area did not show a significant difference at any time point (Figure 3e). These findings indicated that IL‐38 treatment suppressed adventitial ECM remodeling, which may be associated with the reduced incidence of AAA formation. We also assessed elastin degradation, which is caused by MMPs (Dale et al., 2015). The elastin degradation score was significantly reduced in the IL‐38‐treated mice on days 7 and 14 after angiotensin II infusion (Figure 3f). These findings suggest that IL‐38 has protective roles against AAA formation.

### 3.4 IL‐38 reduces MMP‐2 and MMP‐9 production in macrophage in vitro

**FIGURE 4:**
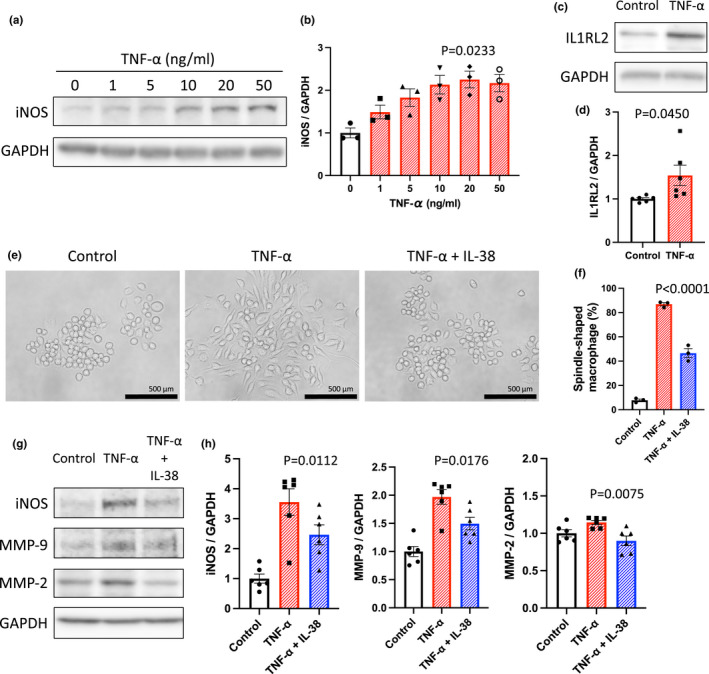
IL‐38 reduced the TNF‐α‐induced production of MMP‐9 and MMP‐2 in macrophages in vitro. (a) Representative western blot analysis of iNOS and GAPDH immunoreactivity in RAW 264.7 cells after TNF‐α stimulation (0, 1, 5, 10, 20, 50 ng/ml). (b) Bar graph showing relative densitometry of iNOS (*n* = 3, *p* = 0.0411 [Kruskal–Wallis test], *p* = 0.0233 [post hoc, 0 vs. 20 ng/ml]). (c) Representative western blot analysis of IL1RL2 and GAPDH immunoreactivity in RAW 264.7 cells after TNF‐α stimulation (20 ng/ml). (d) Bar graph showing relative densitometry of IL1RL2 (*n* = 6, *p* = 0.0450 [*t* test], *p* = 0.0043 [Mann–Whitney U test]). (e) Representative photomicrographs of morphological changes of RAW 264.7 cells after TNF‐α stimulation (20 ng/ml) with or without IL‐38 treatment (100 ng/ml). Scale bar, 500 μm. (f) Bar graph showing the ratio of spindle‐shaped cells among macrophages after TNF‐α stimulation (20 ng/ml) with or without IL‐38 treatment (100 ng/ml; *n* = 3, *p* < 0.0001 [ANOVA], *p* < 0.0001 [post hoc, Control vs. TNF‐α], *p* < 0.0001 [post hoc, Control vs. TNF‐α + IL‐38], *p* < 0.0001 [post hoc, TNF‐α vs. TNF‐α + IL‐38]). (g) Representative western blot analysis of iNOS, MMP‐9, MMP‐2, and GAPDH immunoreactivity in RAW 264.7 cells after TNF‐α stimulation (20 ng/ml) with or without IL‐38 treatment (100 ng/ml). (h) Bar graphs showing the relative densitometry of iNOS (*n* = 6, *p* = 0.0003 [ANOVA], *p* = 0.0016 [post hoc, Control vs. TNF‐α], *p* = 0.0019 [post hoc, Control vs. TNF‐α + IL‐38], *p* = 0.0112 [post hoc, TNF‐α vs. TNF‐α + IL‐38]), MMP‐9 (*n* = 6, *p* = 0.0002 [ANOVA], *p* = 0.0009 [post hoc, PBS vs. TNF‐α], *p* = 0.0470 [post hoc, PBS vs. TNF‐α + IL‐38], *p* = 0.0176 [post hoc, TNF‐α vs. TNF‐α + IL‐38]), and MMP‐2 (*n* = 6, *p* = 0.0097 [ANOVA], *p* = 0.1222 [post hoc, Control vs. TNF‐α], *p* = 0.3385 [post hoc, Control vs. TNF‐α + IL‐38], *p* = 0.0075 [post hoc, TNF‐α vs. TNF‐α + IL‐38]).

Because TNF‐α is associated with MMP‐9 and MMP‐2 production in macrophages (Xiong et al., 2009) and Figure 1 indicated that IL1RL2^+^ macrophages may have important roles in AAA development in vivo, we investigated TNF‐α‐stimulated macrophages in vitro. As expected, iNOS expression was increased after TNF‐α stimulation (Figure 4a,b), indicating that TNF‐α promoted cytokine production in macrophages. IL1RL2 expression was also increased in macrophages after TNF‐α stimulation, similar to the in vivo results (Figure 1e,f, Figure 4c,d). Because IL1RL2 is an IL‐38 receptor, we examined whether IL‐38 affects macrophage activation. Figure 4e shows the morphological changes in macrophages after TNF‐α stimulation in the presence or absence of IL‐38. After TNF‐α stimulation, the number of spindle‐shaped macrophages significantly increased with multiple pseudopodia (Figure 4e, middle panel); however, IL‐38 reduced the number of spindle‐shaped macrophages (Figure 4e, right panel). This result indicated that IL‐38 suppressed TNF‐α‐induced macrophage activation. To examine the effects of IL‐38 on macrophage function, we examined iNOS, MMP‐2, and MMP‐9 expression in TNF‐α‐stimulated macrophages. Western blot analysis showed that IL‐38 treatment significantly suppressed the increase in iNOS, MMP‐9, and MMP‐2 expression induced by TNF‐α (Figure 4f,g). These findings suggest that IL‐38 may be able to reduce MMP‐9 and MMP‐2 production in macrophages.

### 3.5 IL‐38 suppresses AAA formation in a p38 phosphorylation‐dependent manner

**FIGURE 5:**
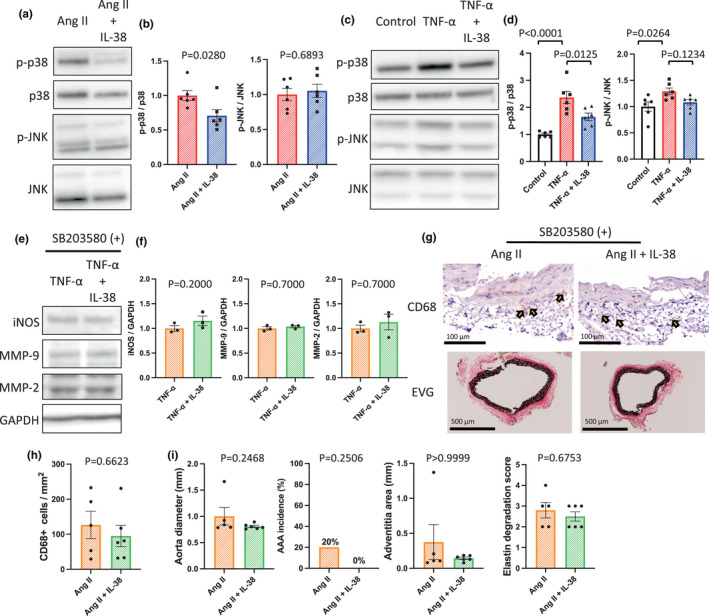
The mechanism how interleukin‐38 suppresses macrophage activation. (a) Representative western blot analysis of p‐p38, p38, p‐JNK, and JNK in the aortic wall on day 14. (b) Bar graphs showing the relative densitometry of p‐p38 (*n* = 6, *p* = 0.0280 [*t* test], *p* = 0.0411 [Mann–Whitney U test]) and p‐JNK (*n* = 6, *p* = 0.6893 [*t* test], *p* = 0.6991 [Mann–Whitney U test]). (c) Representative western blot analysis of p‐p38, p38, p‐JNK, and JNK in macrophages after TNF‐α stimulation with or without IL‐38. (d) Bar graphs showing the relative densitometry of p‐p38 (*n* = 6, *p* < 0.0001 [ANOVA], *p* < 0.0001 [post hoc, Control vs. TNF‐α], *p* = 0.0234 [post hoc, Control vs. TNF‐α + IL‐38], *p* = 0.0125 [post hoc, TNF‐α vs. TNF‐α + IL‐38]) and p‐JNK (*n* = 6, *p* = 0.0283 [ANOVA], *p* = 0.0264 [post hoc, Control vs. TNF‐α]). (e) Representative western blot analysis of iNOS, MMP‐9, MMP‐2, and GAPDH in macrophages after TNF‐α stimulation with or without IL‐38 in the presence of SB203580. (f) Bar graphs showing the relative densitometry of iNOS (*n* = 3, *p* = 0.2000 [Mann–Whitney U test]), MMP‐9 (*n* = 3, *p* = 0.7000 [Mann–Whitney U test]), and MMP‐2 (*n* = 3, *p* = 0.7000 [Mann–Whitney U test]). (g) Representative photomicrographs of the aortic wall on day 14 in the presence of SB203580, stained with EVG (upper row) or anti‐CD68 antibody (lower row). (h) Bar graph showing the number of CD68^+^ cells in the aortic wall on day 14 in the presence of SB203580 (*n* = 5–6, *p* = 0.6623 [Mann–Whitney U test]). (i) Bar graphs showing the aorta diameter (*n* = 5–6, *p* = 0.2468 [Mann–Whitney U test]), AAA incidence (*n* = 5–6, *p* = 0.2506 [χ^2^ test]), adventitia (*n* = 5–6, *p* > 0.9999 [Mann–Whitney U test]), intramedia (*n* = 5–6, *p* = 0.1710 [Mann–Whitney U test]), and elastin degradation score (*n* = 5–6, *p* = 0.4917 [*t* test], *p* = 0.6753 [Mann–Whitney U test]) on day 14 in the presence of SB203580.

Our results indicated that IL‐38 reduced MMP‐9 and MMP‐2 production in the aortic wall (Figure 2) to downregulated AAA formation (Figure 3). To determine how IL‐38 suppresses macrophage activation and AAA formation, we examined the p38 and JNK signaling pathways, which are associated with MMP‐9 and MMP‐2 production. IL‐38 treatment significantly suppressed p38 phosphorylation both in vivo and in vitro (Figure 5a–d); however, it did not suppress JNK phosphorylation either in vivo or in vitro (Figure 5a–d). These results indicated that IL‐38 regulation of AAA formation depends on the p38 signaling pathway. To further understand the mechanism, we used SB203580 to inhibit p38 phosphorylation. If IL‐38 treatment depends on p38 phosphorylation, SB203580 should diminish the effects of IL‐38 during AAA formation. As expected, SB203580 diminished the IL‐38 effects on iNOS, MMP‐9, and MMP‐2 production (Figure 5e,f). Furthermore, SB203580 reduced the IL‐38 effects on macrophage accumulation (Figure 5g, upper panel, and 5h), aorta diameter, and adventitia area (Figure 5i), which were suppressed by IL‐38 (Figures 2, 3). In summary, IL‐38 reduced macrophage accumulation in the aortic wall during AAA formation (Figure 2a–d) to regulate MMP‐9 and MMP‐2 production (Figure 2e–g) and suppress AAA formation (Figure 3) in a p38‐dependent manner (Figure 5).

## 4 DISCUSSION

The present study showed that IL1RL2^+^ macrophage accumulation along with MMP‐2 and ‐9 expression was associated with angiotensin II‐induced mouse AAA formation (Figure 1). IL‐38 treatment suppressed macrophage activities (Figure 4) and M1 macrophage accumulation in remodeling the aortic wall (Figure 2a–d) with lower MMP‐2 and ‐9 expression (Figure 2e–g), which reduced the AAA incidence (Figure 3) in angiotensin II‐induced AAA in C57BL6/J mice. We also revealed that IL‐38 regulated macrophages to reduce the AAA incidence in a p38 pathway‐dependent manner (Figure 5).

AAA rupture should be prevented to avoid death, however, only surgical treatments have been recommended by the latest guidelines (Chaikof et al., 2018). Several medical therapies have been considered, however, none of them can substitute for surgery (Bicknell et al., 2016; Chaikof et al., 2018; Thompson et al., 2010). Macrophages play key roles in AAA formation (Raffort et al., 2017), hence we focused on IL‐38, which suppresses macrophage activities. IL‐38 treatment reduced M1 macrophage accumulation in the aortic wall (Figure 2a–d), which reduced the incidence of AAA formation (Figure 3), indicating that IL‐38 treatment may be a novel therapy for AAA patients.

Veerdonk et al. have reported that IL‐38 bound only to IL1RL2 (van de Veerdonk et al., 2012). Because IL1RL2 regulates inflammation (Buhl & Wenzel, 2019), IL‐38 may control inflammation specifically through IL1RL2 after angiotensin II induction. We observed IL1RL2^+^ macrophage accumulation (Figure 1d–f) along with increased TNF‐α, MMP‐2, and MMP‐9 expression (Figure 1a–c). Consistently, a previous study has shown that IL1RL2 expression increased in mouse macrophages under inflammatory conditions (Ge et al., 2021). As expected, IL‐38 suppressed macrophage accumulation and MMP‐2 and ‐9 expression in the aortic wall during AAA formation (Figure 2).

We further showed that IL‐38 suppressed only M1 macrophage accumulation in the aortic wall (Figure 2c,d). M1 macrophages have been shown to participate in the AAA development by enhancing vascular inflammation and inducing elastin degradation (Dale et al., 2015). IL‐38 suppressed M1 macrophage accumulation in the aortic wall, and thus may reduce the AAA incidence by regulating M1 macrophages. Previous reports have shown that M1 macrophages precede M2 macrophages after angiotensin II infusion (Raffort et al., 2017; Rateri et al., 2011), indicating that inflammation precedes tissue remodeling during AAA formation. IL‐38 suppressed the accumulation of M1 macrophages, but not M2 macrophages (Figures 2, 4), indicating that IL‐38 suppressed inflammation, while the tissue remodeling effects of macrophages were maintained. Because the inflammatory response increases MMP‐2 and ‐9 to promote elastin degradation (Longo et al., 2002), IL‐38 may also suppress MMP‐2 and ‐9 expression (Figure 2e–g) to reduce elastin degradation (Figure 3f) by regulating macrophages. These protective effects of IL‐38 for remodeling the aortic wall may reduce the AAA incidence and improve the survival of the mouse angiotensin II‐induced AAA model.

We also demonstrated that the protective effects of IL‐38 for remodeling the aortic wall depend on the p38 signaling pathway in angiotensin II‐induced AAA model mice (Figure 5). A previous report has shown that IL‐38 alleviated the common virus infection‐induced cytokine production through the intracellular STAT1, STAT3, p38, ERK1/2, MEK, and NF‐κB signaling pathways in vitro (Gao, Chan, et al., 2021). In this study, we focused on the p38 and JNK signaling pathways because mounting evidence suggests that p38 and JNK are activated in AAA patients and mice, and the p38 and JNK pathways are associated with MMP‐9 and MMP‐2 production (DiMusto et al., 2012; Dodd et al., 2011; Gao, Gao, et al., 2021; Yoshimura et al., 2005). We revealed that IL‐38 inhibited p38 phosphorylation both in vitro and in vivo, but not JNK (Figure 5a–d). To further assess whether IL‐38 depends on the p38 signaling pathway or not, we used SB203580 to inhibit p38 phosphorylation. Inhibition of p38 phosphorylation diminished the protective effects of IL‐38 on MMP‐2 and ‐9 production (Figure 5e,f), macrophage accumulation (Figure 5g,h), and the AAA incidence (Figure 5i). These findings indicated the protective effects of IL‐38 for remodeling the aortic wall depend on the p38 signal pathway.

In conclusion, IL‐38 plays protective roles in AAA formation through regulation of M1 macrophage accumulation and MMP‐2 and ‐9 expression in the aortic wall. Therefore, IL‐38 treatment may become a novel medical therapy for AAA patients.

## AUTHORS' CONTRIBUTIONS

Shun Kurose and Yutaka Matsubara conceived the idea of the study. Shun Kurose and Yutaka Matsubara developed the statistical analysis plan and conducted statistical analyses. Shun Kurose, Yutaka Matsubara, Shinichiro Yoshino, Keiji Yoshiya, Koichi Morisaki, Tadashi Furuyama, and Tomoaki Hoshino contributed to the interpretation of the results. S.K. drafted the original manuscript. Tadashi Furuyama and Tomoharu Yoshizumi supervised the conduct of this study. All authors reviewed the manuscript draft and revised it critically on intellectual content. All authors approved the final version of the manuscript to be published.

## FUNDING INFORMATION

No funding information provided.

## Supporting information


Figure S1.
Figure S2.
Figure S3.
Figure S4.
Figure S5.
