# Supplementary material for: Interleukin‐38 suppresses abdominal aortic aneurysm formation in mice by regulating macrophages in an IL1RL2‐p38 pathway‐dependent manner
Source: Physiol Rep. 2023 Jan 28;11(2):e15581. doi: 10.14814/phy2.15581 (PMC9884112; doi:10.14814/phy2.15581)
Supplement: Supplementary file 1 — Figure S1. Figure S2. Figure S3. Figure S4. Figure S5. [file PHY2-11-e15581-s001.pdf]

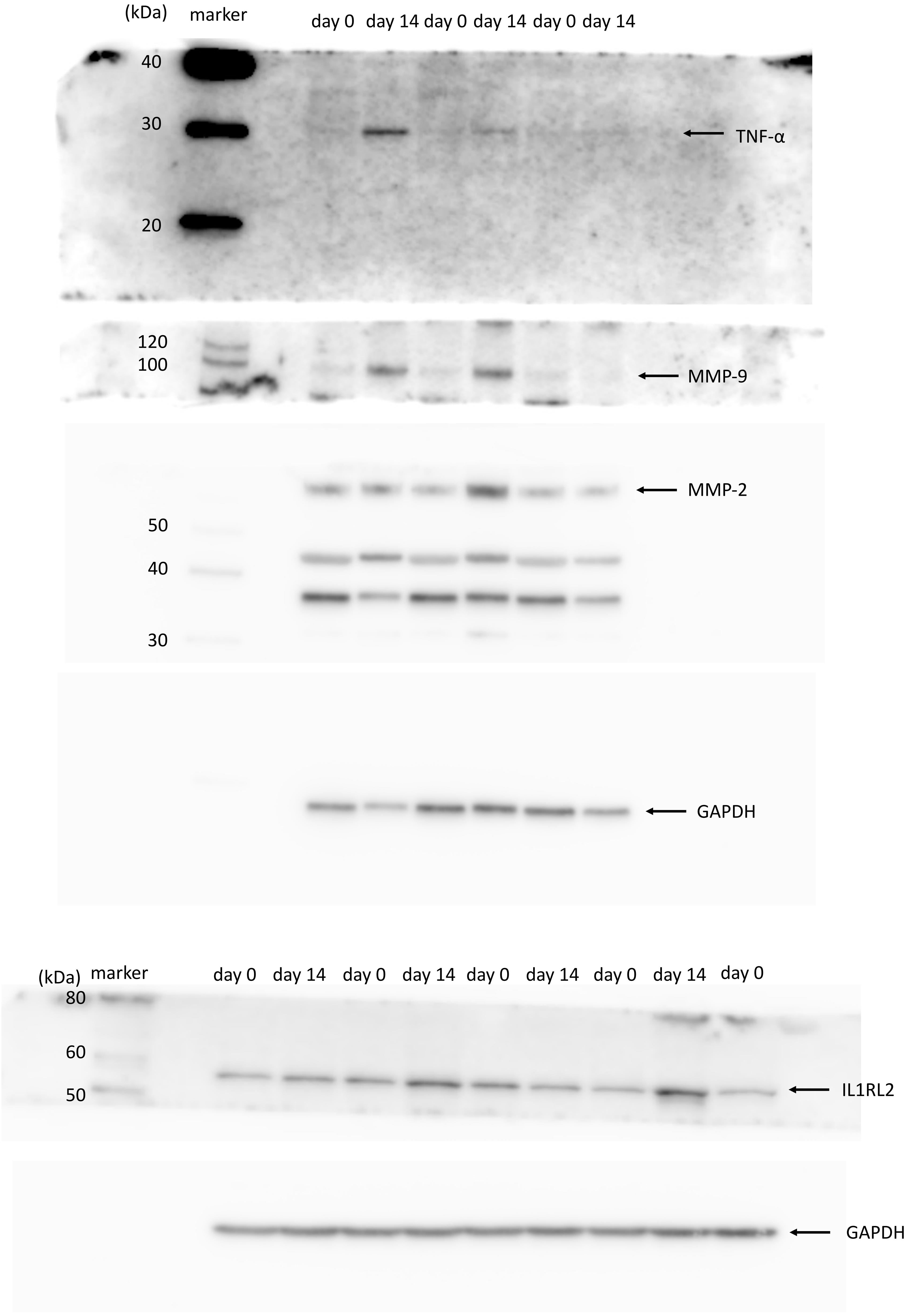

Supplementary Figure S1. Uncropped blots probed with TNF-α, MMP-9, MMP-2, IL1RL2 and GAPDH as presented in Figure 1B and 1E

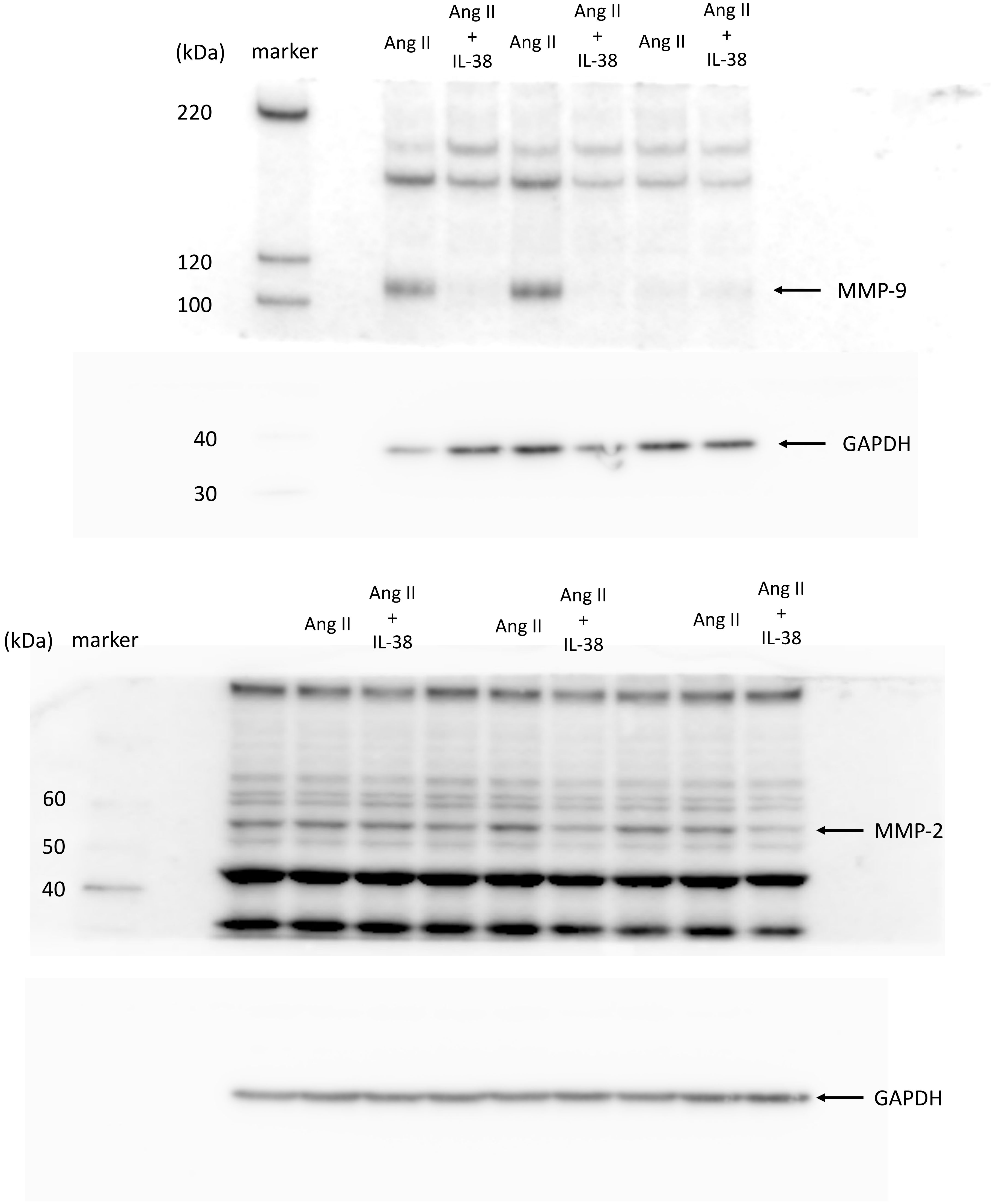

Supplementary Figure S2. Uncropped blots probed with MMP-9, MMP-2 and GAPDH as presented in Figure 2F

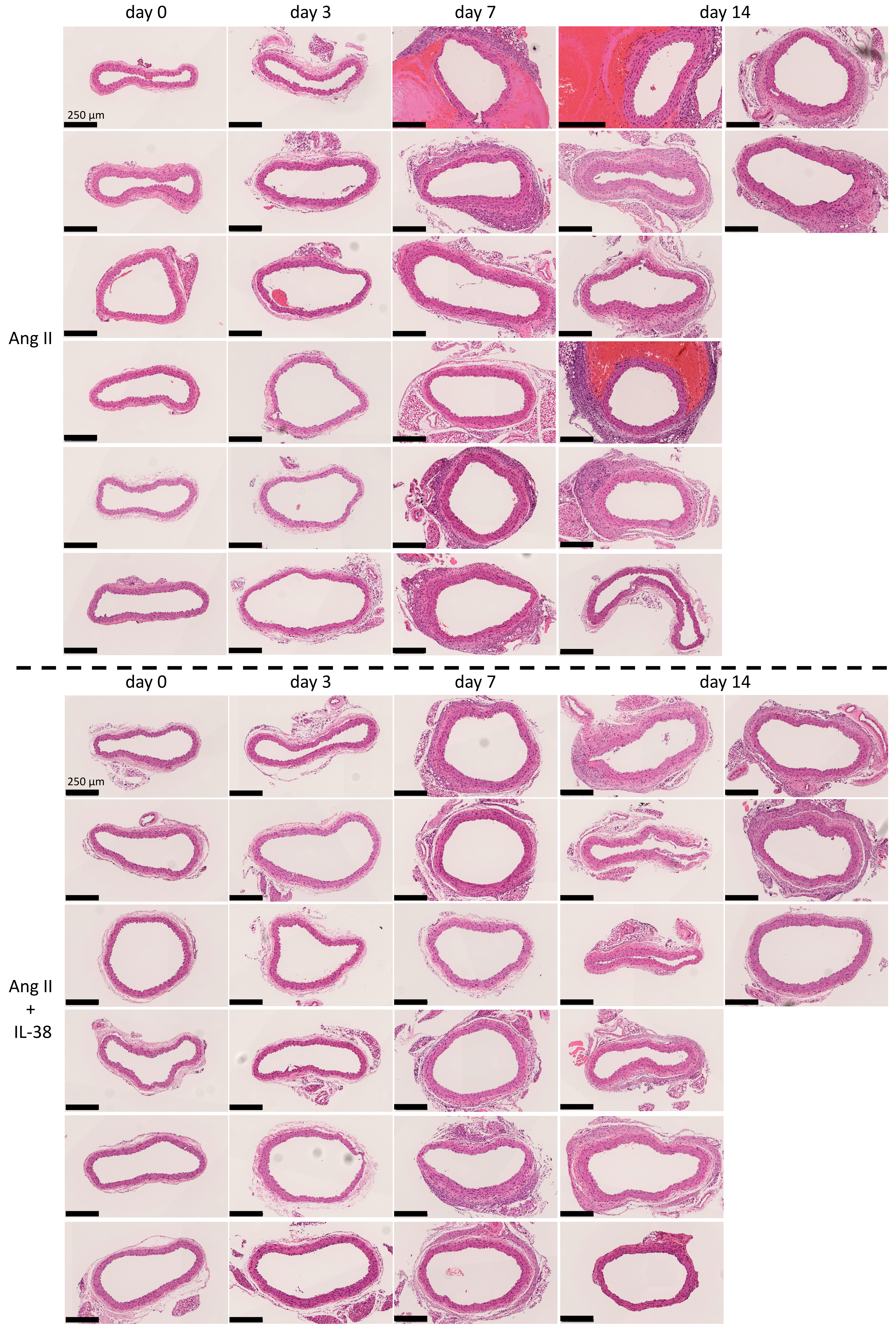

Supplementary Figure S3. All photomicrographs of the aortic wall stained with H&E as presented in Figure 3A; Scale bar, 250 µm

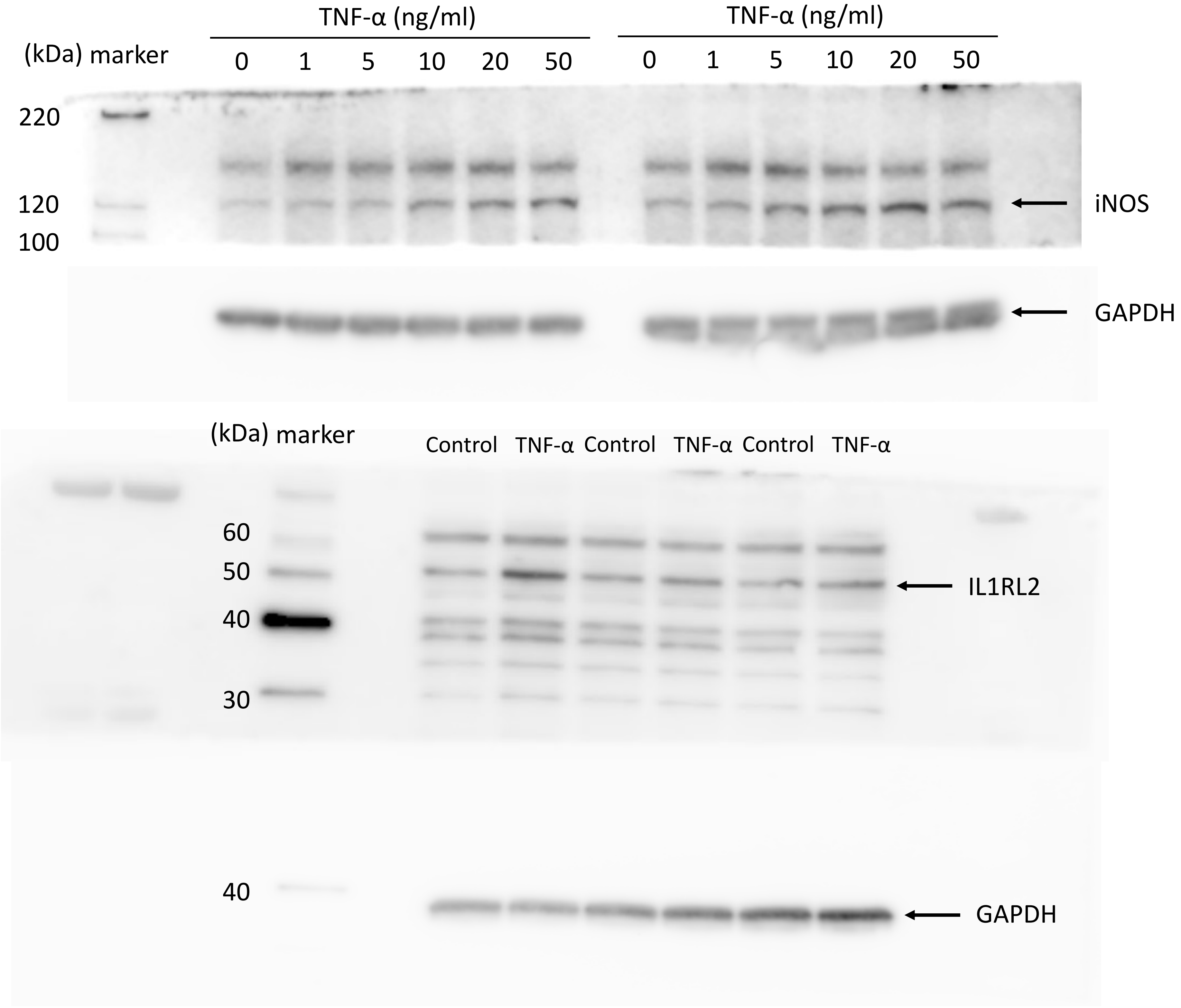

Supplementary Figure S4a. Uncropped blots probed with IL1RL2, iNOS and GAPDH as presented in Figure 4A and 4C

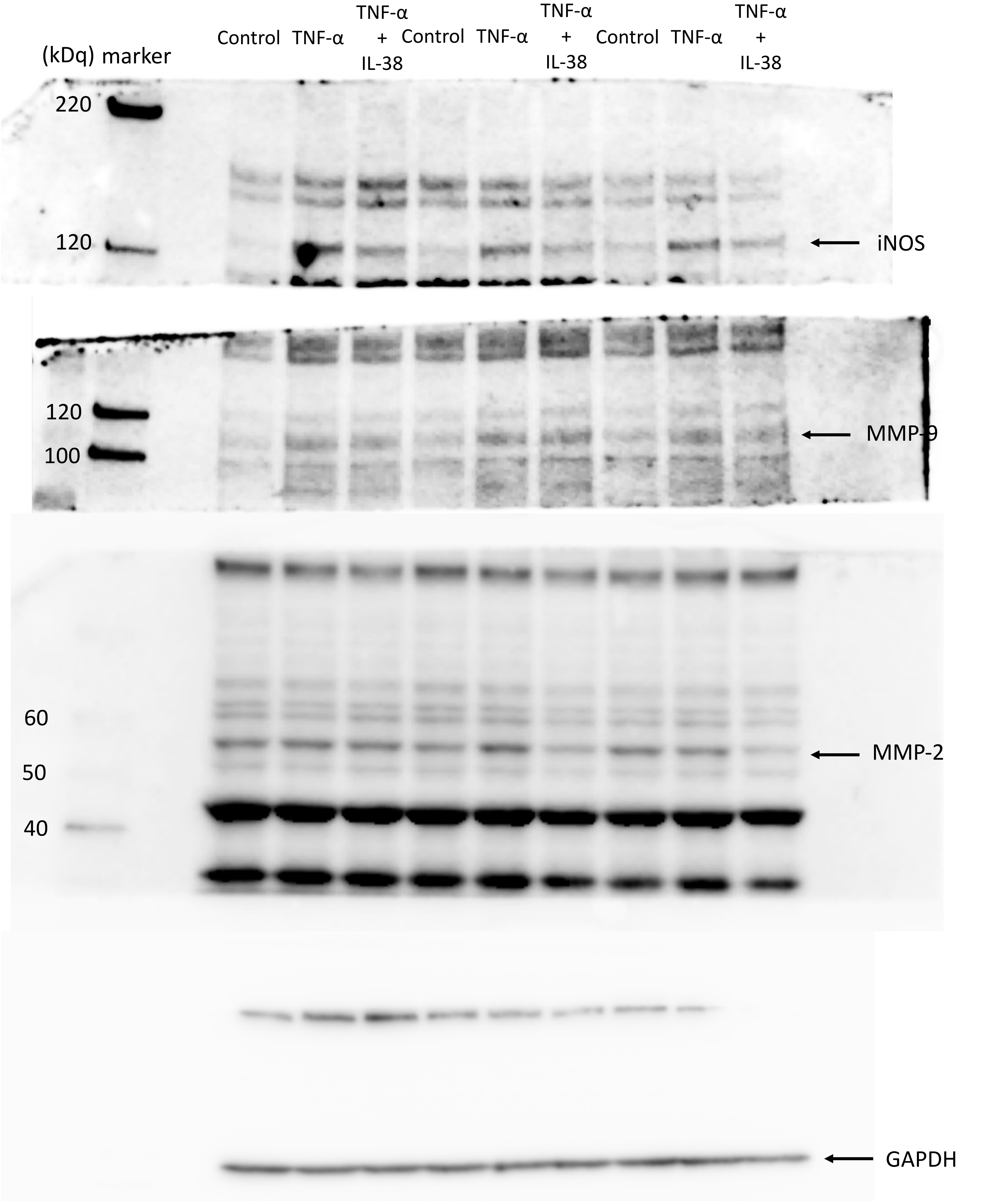

Supplementary Figure S4b. Uncropped blots probed with iNOS, MMP-9, MMP-2 and GAPDH as presented in Figure 4G

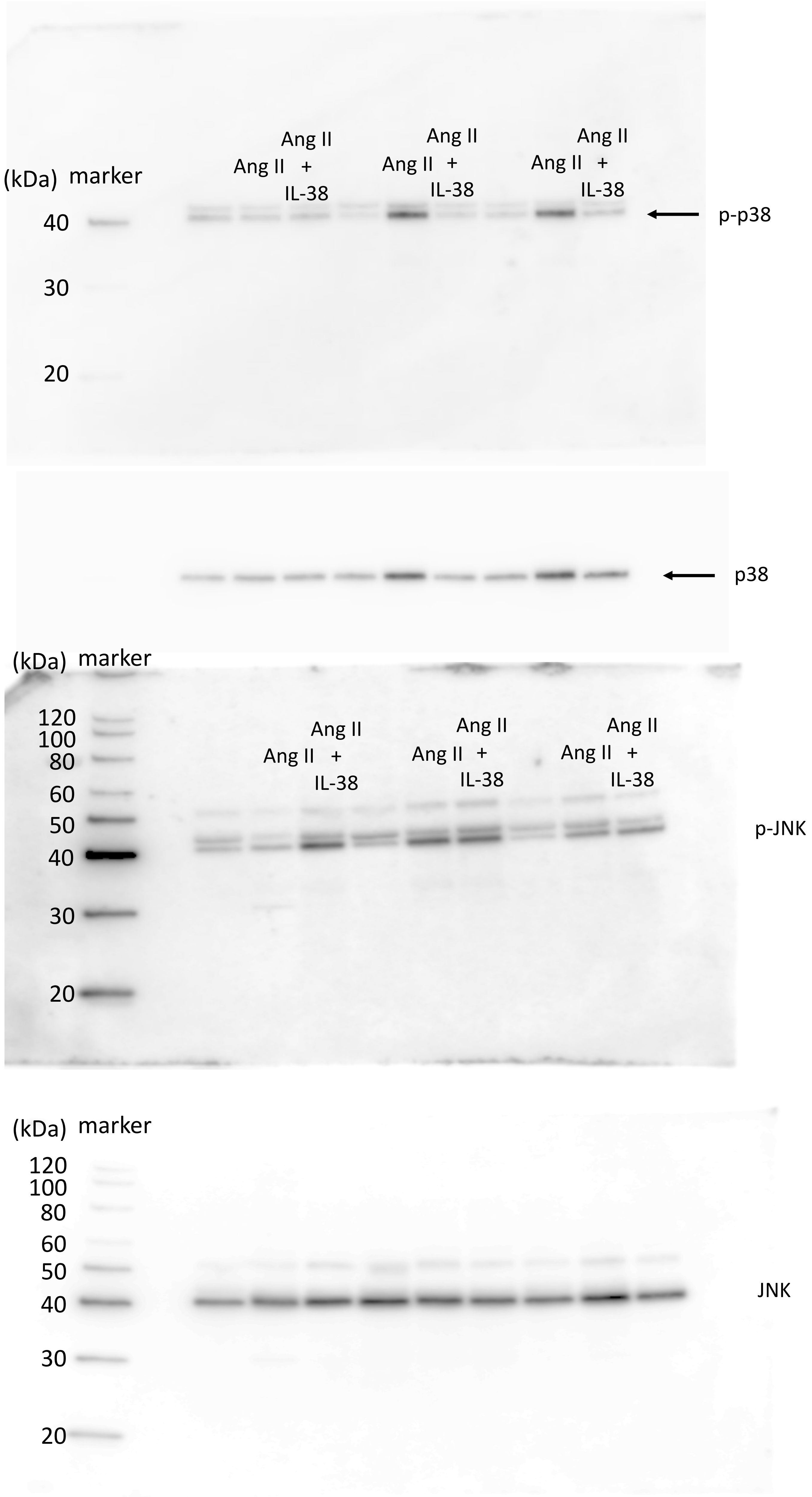

Supplementary Figure S5a. Uncropped blots probed with p-38, p-p38, JNK and p-JNK as presented in Figure 5A

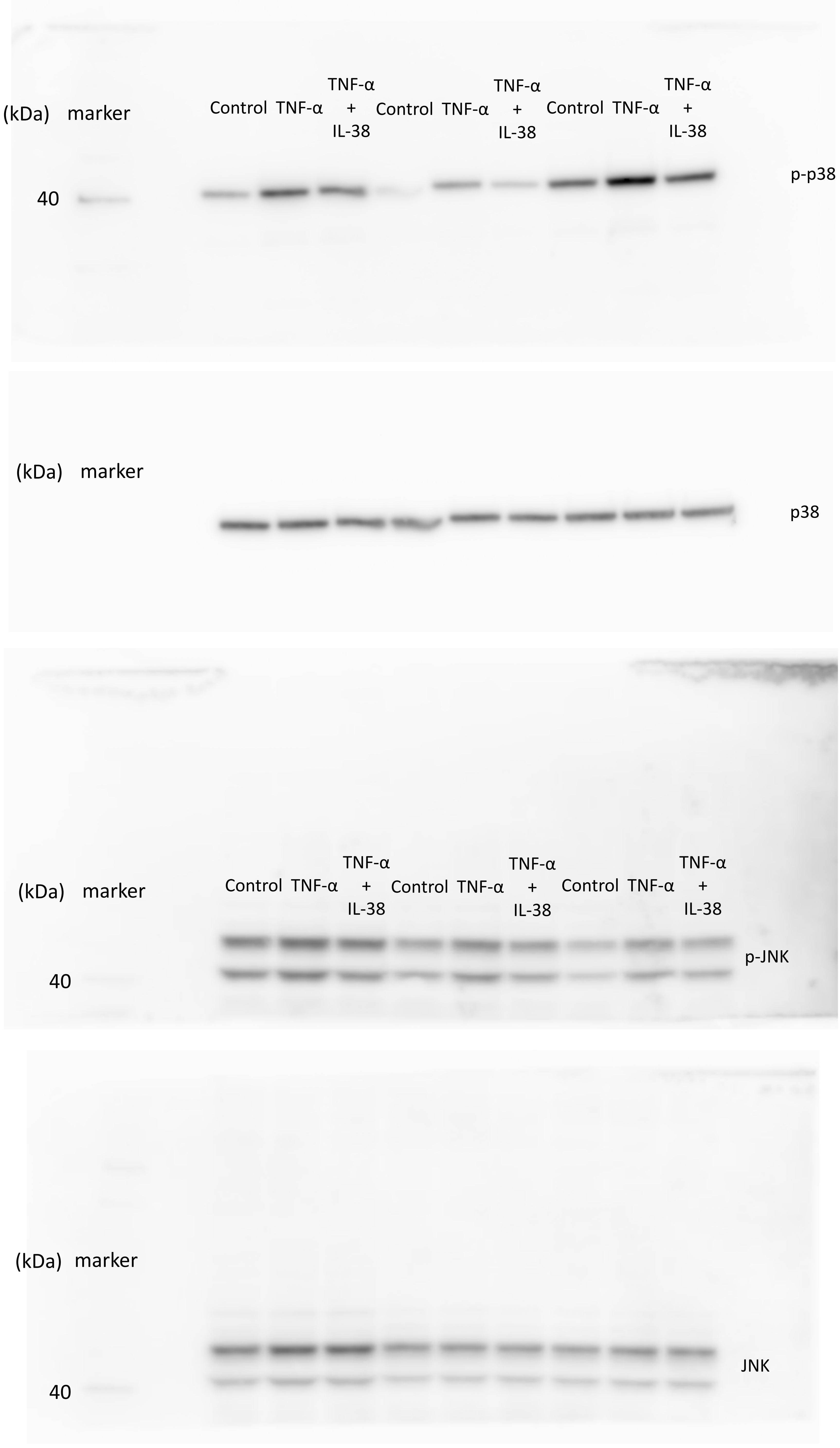

Supplementary Figure S5b. Uncropped blots probed with p-38, p-p38, JNK and p-JNK as presented in Figure 5C

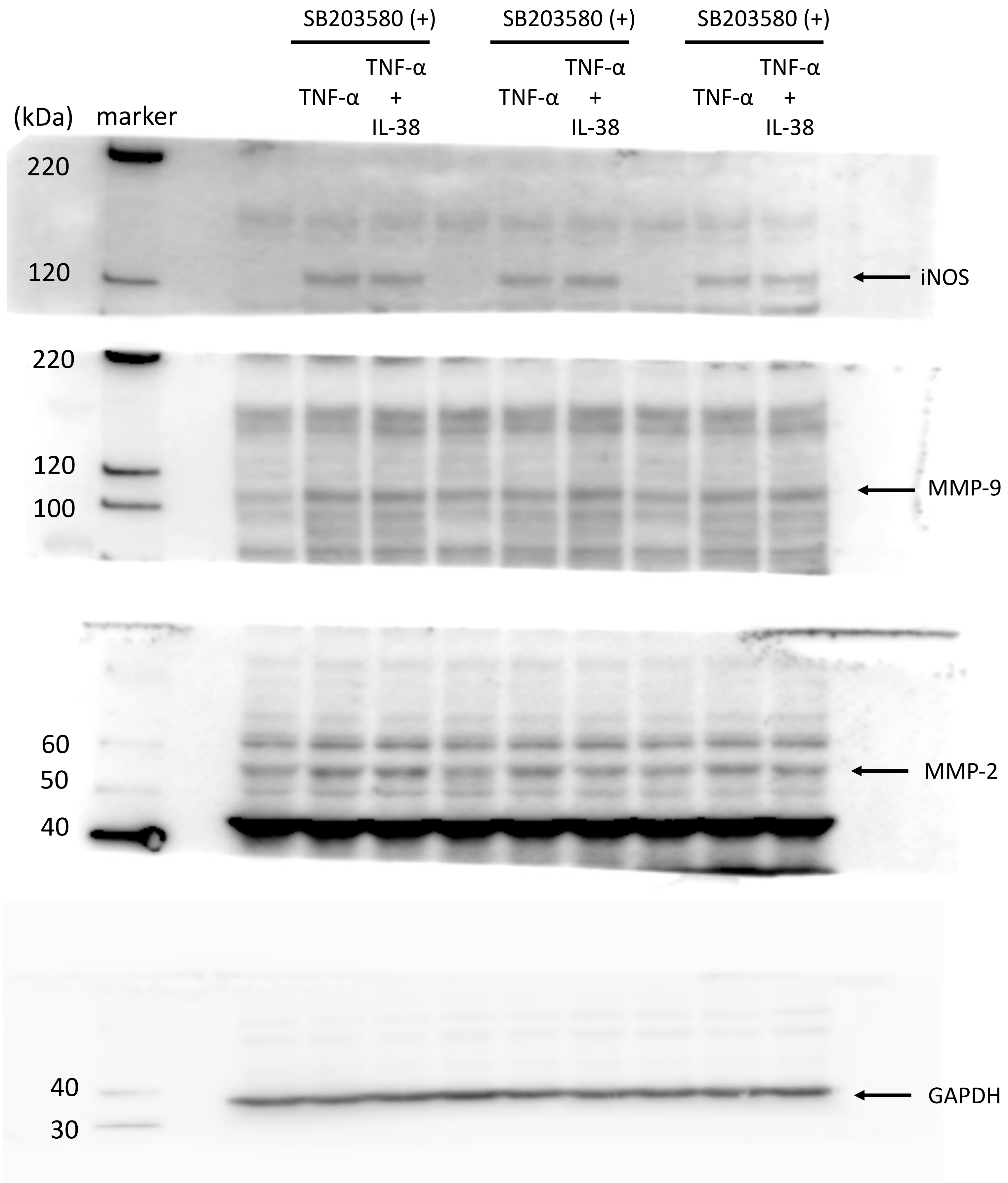

Supplementary Figure S5c. Uncropped blots probed with iNOS, MMP-9, MMP-2 and GAPDH as presented in Figure 5E
